# Adapting the Bogenhausen Dysarthria Scales (BoDyS) to Chilean Spanish Speakers: Face and Content Validation

**DOI:** 10.3390/brainsci15060604

**Published:** 2025-06-04

**Authors:** Marcela Sanhueza-Garrido, Virginia García-Flores, Sebastián Contreras-Cubillos, Jaime Crisosto-Alarcón

**Affiliations:** Departamento de Ciencias de la Rehabilitación en Salud, Facultad de Ciencias de la Salud y los Alimentos, Universidad del Bío-Bío, Chillán 3800708, Chile; mlsanhueza@ubiobio.cl (M.S.-G.); vgarcia@ubiobio.cl (V.G.-F.); scontreras@ubiobio.cl (S.C.-C.)

**Keywords:** speech assessment, neuromotor speech disorders, dysarthria evaluation, cross-cultural adaptation, cross-linguistic validation

## Abstract

**Background:** Dysarthria is a neuromotor speech disorder that significantly impacts patients’ quality of life. In Chile, there is a lack of culturally validated instruments for assessing dysarthria. This study aimed to cross-culturally adapt the Bogenhausen Dysarthria Scales (BoDyS) into Chilean Spanish and to conduct face and content validation. **Methods:** The adaptation process included translation and back-translation, followed by validation by a panel of experts. Clarity, format, and length were evaluated, and the Kappa index (KI), content validity index (CVI), and content validity ratio (CVR) were calculated to confirm item relevance. A pilot test was subsequently conducted with ten speech–language pathologists to apply the adapted version to patients. **Results:** The adaptation process produced a consensus version that preserved the semantic and cultural characteristics of the original scale. The statistical measures (KI = 1.00; I-CVI = 1.00; S-CVI/Ave = 1.00; S-CVI/UA = 1.00; CVR = 1.00) indicated satisfactory levels of agreement. The pilot test demonstrated the scale’s appropriateness and effectiveness for assessing dysarthria within the Chilean context, although some experts recommended reducing task repetition for patients prone to fatigue. **Conclusions:** The Chilean version of the BoDyS (BoDyS-CL) is a valid and useful tool for evaluating dysarthria in Chile. This study provides a foundation for further research and the systematic implementation of this scale in local clinical practice.

## 1. Introduction

Dysarthria is a group of neurogenic speech disorders characterized by abnormalities in the strength, speed, range, steadiness, tone, or accuracy of movements required for respiration, phonation, resonance, articulation, and prosody, all of which enable speech production [1]. This speech disorder is caused by damage to the areas of the central or peripheral nervous system that control the muscles responsible for speech production [1]. According to Duffy (2013), the pathogenesis of dysarthria can be classified according to the site of injury, resulting in different types of dysarthria, such as spastic, flaccid, ataxic, hypokinetic, and hyperkinetic. Damage to cerebral motor areas, such as the motor cortex, basal ganglia, cerebellum or nerve pathways, affects coordination, strength, and speech control. The severity and specific symptoms depend on the type and location of the lesion [2].

Clinical manifestations include alterations in articulation, rhythm, pitch, and intensity of speech [2]. These include slow or rapid speech, imprecision in the articulation of words, and alterations in prosody, such as monotony and increased or decreased muscle tone (spasticity or flaccidity) that affects breathing and vocal control [3]. The speech difficulties associated with dysarthria can significantly hinder communication and daily activities, impacting psychosocial dynamics and diminishing the patient’s overall quality of life [4,5]. Additionally, depending on the underlying cause of dysarthria, it may coexist with other neurogenic disorders affecting language, cognition, and/or swallowing [6]. To comprehensively assess a patient’s condition, it is crucial to conduct functional assessments using standardized methodologies. This approach facilitates the diagnostic process and supports the development of tailored therapeutic plans that align with the unique needs of each person.

Currently, there is no consensus on the most appropriate method for assessing dysarthria or the specific aspects that should be considered. However, a comprehensive initial speech assessment generally should include the following components: (1) a thorough clinical history, (2) an examination of the speech mechanism and oral motor skills, (3) an assessment of the speech subsystems (including respiration, phonation, articulation, resonance, and prosody), (4) a perceptual assessment, and (5) an evaluation of intelligibility [7]. The American Speech–Language–Hearing Association also suggests that functional speech characteristics, such as naturalness, efficiency, and intelligibility, should be integral to the assessment process [8]. Furthermore, it is crucial to evaluate the impact of dysarthria on the patient’s activities and participation, as this helps assess the disorder’s social and psycho-emotional effects on the person [5,8,9].

The evidence comparing assessment instruments for dysarthria indicates several key issues: (1) a lack of standardization regarding the aspects evaluated, scoring criteria, and administration procedures; (2) a limited scope, where some protocols concentrate solely on articulation or prosody, while others prioritize intelligibility or functional communication; (3) insufficient sensitivity, failing to account for the cultural and linguistic diversity of individuals with dysarthria; (4) notable limitations in assessing quality of life; and (5) the necessity of implementing assessment tools that extend beyond just speech components [10,11,12,13,14]. Furthermore, a preference for informal assessment tools over standardized ones has been noted, particularly in Spanish-speaking countries, where standardized protocols are often unavailable [14]. Additionally, Chilean Spanish’s phonetic and phonological characteristics [15,16] may affect how speech intelligibility and naturalness are perceived in individuals with dysarthria. Therefore, cultural and linguistic adaptation of any assessment tool are crucial to ensure its applicability and accuracy.

Currently, the predominant framework for diagnosing dysarthria is a classification system developed at the Mayo Clinic, grounded in the perceptual characteristics of the disorder [17,18,19]. These characteristics aid in identifying the underlying neurological conditions associated with this speech impairment. However, it is important to note that this approach may have limitations due to the numerous variables involved [20]. Assessment protocols such as the Frenchay Dysarthria Assessment-2 [21] and the methods proposed by Duffy [2] emphasize the importance of using a broad range of assessment tasks, enabling therapists to better understand their patients’ speech disorders. However, the significant inter-rater variability in clinical assessments can pose challenges in this process [20]. In this context, the Bogenhausen Dysarthria Scales (BoDyS) [20] offer a promising alternative by providing a standardized measurement of clinically relevant dimensions of speech affected by dysarthria.

The BoDyS employs speech tasks exclusively for assessment. These tasks include open-ended questions to elicit spontaneous speech, sentence repetition, reading short texts, and storytelling from images, resulting in 12 audio-visual speech samples for later analysis. The application is adaptable to meet the clinical needs of each patient, offering three versions: the complete version, which encompasses all four modalities and twelve tasks; an abbreviated version that optimizes time by reducing the number of tasks; and a version without reading, tailored for patients with visual or cognitive challenges. Assessments typically last between 15 and 30 min, with subsequent analysis taking 20 to 40 min, depending on the examiner’s expertise. The BoDyS assesses nine dimensions of speech and demonstrates high inter-rater reliability, with indices exceeding 0.85, alongside empirical validity verified by factor analysis. Its practical approach facilitates treatment planning and monitoring progress or recovery, while auditory perception aids therapeutic intervention. This flexibility and clinical robustness combination makes the BoDyS a valuable tool for assessing dysarthria in clinical and research contexts [20].

In Chile, the work conducted at the Mayo Clinic has significantly contributed to the advancement of the field, leading to the development of instruments such as the Speech Assessment Protocol [22] and the Speech Assessment Protocol for Dysarthria (PEV-H-Disartria for its acronym in Spanish) [23]. These tools incorporate the assessment of neuromotor aspects of speech, as well as the assessment of various speech subsystems and their functional characteristics. However, Chile currently lacks standardized instruments for assessing speech in individuals with dysarthria, instruments that would allow for assessing their performance over time and tracking progress in treatment through speech-only tasks [14]. Based on the above, there is a need for a culturally and linguistically adapted dysarthria assessment tool adapted to Chilean Spanish, which will improve the accuracy of diagnosis, treatment planning, and progress monitoring in the rehabilitation of patients with dysarthria in Chile.

Considering the above, this study aims to carry out the cross-cultural adaptation to Chilean Spanish and the face and content validation of the Bogenhausen Dysarthria Assessment Scales (*Bogenhausener Dysarthrieskalen,* BoDyS) [20]. To our knowledge, this is the first investigation to assess the validity of the BoDyS in a language other than German. This adaptation will provide a tool tailored to the Chilean context, enabling professionals to effectively assess speech in individuals with dysarthria using stimuli, parameters, and rubric-based scoring scales relevant to the local context.

## 2. Materials and Methods

**Figure 1:**
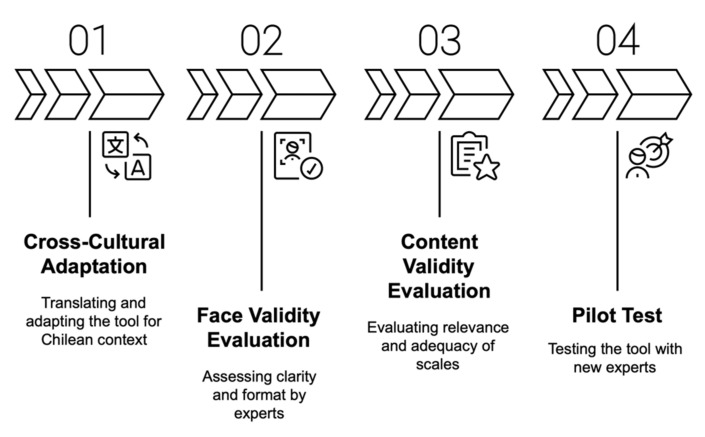
Flowchart of the four methodological stages: (1) translation and cultural adaptation; (2) expert assessment of face validity; (3) expert assessment of content validity; and (4) pilot testing.

The methodological process was carried out in four sequential stages, encompassing the translation and adaptation of the BoDyS, expert validation, pilot testing, and final adjustments. These stages are summarized in Figure 1.

In its original German version, the BoDyS is a standardized assessment tool designed to assess dysarthria in adults. It allows for assessing the overall severity of dysarthria and provides a comprehensive overview of the characteristics of the disorder by generating a detailed profile [24]. This test relies solely on speech tasks, employing auditory analysis methods that emphasize the functional aspects of motor speech performance. The BoDyS comprises four tasks: spontaneous speech, oral repetition, reading aloud, and describing sequences from images. Each task is repeated three times with different stimuli, resulting in a total of 12 speech samples to be analyzed by the evaluator. The evaluation utilizes nine scales that address the primary dimensions of dysarthric impairment: respiration, voice level, voice quality, voice stability, articulation, resonance, articulation rate, fluency, and prosodic modulation. Each speech sample is assessed on these nine scales, receiving a score from 0 to 4, where a score of 4 indicates no impairment and a score of 0 represents the most severe level of impairment [20]. Detailed characteristics of each scale are presented in Table 1.

### 2.1. Stage 1: Cross-Cultural Adaptation Procedure

To ensure the cross-cultural adaptation of the instrument in its complete version for this stage of work, we employed the back-translation methods proposed by Ramada-Rodilla [25] and Wild et al. [26]. Initially, two certified native Chilean translators independently translated the scales and the stimuli manual from German into Spanish. In collaboration with field experts, the research team then developed a consensus version based on these translations. This preliminary consensus version was subsequently presented to three Chilean speech–language pathologists, who evaluated its comprehensibility, the appropriateness of the vocabulary, and the relevance of the translated material. The professionals selected for this task met the following inclusion criteria: (a) they were licensed speech–language pathologists, (b) they had a minimum of five years of professional experience, and (c) they possessed at least three years of experience in speech assessment and intervention. Following this evaluation, the consensus version underwent back-translation into the original language conducted by a professional Spanish–German translator who was a native German speaker. This step aimed to compare the original version of the BoDyS protocol with the back-translated version, identifying any similarities or potential discrepancies in content or vocabulary arising from the modifications.

Specifically, to evaluate the translation of the stimuli manual, the translation was submitted to a professional linguist, who had to adapt the stimuli of the assessment tasks, considering semantic, phonological, and lexical aspects pertinent to Chilean Spanish. It is important to mention that the stimuli used in the BoDyS-CL incorporated a syllabic metric for the stimulus words, like the German version. Likewise, the length and types of sentences (affirmative, exclamatory, and interrogative) have the same characteristics as the original version. Regarding the texts created for the BoDyS-CL, similar themes to those addressed in the German version were considered. For example, one of the texts in the original version is about a traditional German character, the “carpet cleaner.” In contrast, a culturally equivalent character was considered in the Spanish version: the “knife sharpener.” The entire process of adapting the stimuli was evaluated and guided by a team of speech–language pathologists, a translator, and a linguist.

Additionally, it is important to note that new images were created, distinct from the original German version, to align with the Chilean cultural context while preserving the number of scenes in each sequence.

At this stage of the study, the potential inter-dialectal applicability of the instrument across Spanish-speaking populations has not yet been examined. This is because the phrases, texts, and images were developed with reference to the cultural context of Chile. Nevertheless, future stages may involve the adaptation of stimuli to ensure their suitability and relevance for other varieties of Spanish.

### 2.2. Stage 2: Procedure for Face Validity

Both the face and content validity were evaluated by a panel of experts consisting of ten Chilean speech–language pathologists (eight women and two men), who were selected through convenience sampling and in accordance with the criteria set forth by Cabero and Barroso [27]. These criteria allow for the calculation of the so-called ‘expert competence coefficient’ or ‘K coefficient’, which is derived from a self-assessment conducted by the individual to determine their expert competence in the research subject. It is calculated using the following formula: K = 1/2 (Kc + Ka), where Kc is the ‘knowledge coefficient’, representing the expert’s knowledge about the given topic or problem, and Ka is the ‘argumentation coefficient’, which refers to the reasoning behind the expert’s criteria [27].

All panel members had at least five years of experience and worked in different health centers in Chile to care for patients with neuromotor speech disorders.

The BoDyS underwent a face validity evaluation process. These scales consist of the stimuli manual, a description of the scales and their features, and the recording sheet used to note the frequency of occurrence of dysarthria characteristics, also referred to as features, for each scale. Each component of the BoDyS was assessed using three criteria: (1) clarity, which examines whether the text is clear and possesses appropriate semantic and syntactic qualities; (2) format, which evaluates whether the presentation of information, images, font, and font size are suitable; and (3) length, which considers whether the length of the texts is appropriate and facilitates the reader’s understanding. These criteria were rated on a 5-point Likert scale, with 1 indicating ‘strongly disagree’ and 5 signifying ‘strongly agree’.

### 2.3. Stage 3: Procedure for Content Validity

Content validity refers to the extent to which a measurement instrument encompasses the various dimensions of the concept being assessed. An instrument is deemed content-valid if it effectively addresses all relevant aspects of the concept under investigation [28]. Both qualitative and quantitative approaches were used to evaluate the content validity of the instrument [29,30].

In this stage, the same ten expert speech–language pathologists who participated in the face validity assessment were involved. Participants were tasked with completing a rubric to assess the clarity and relevance of each item, as well as the adequacy of the scales. They used Likert-type indicators rated from 1 to 4 to assess each criterion. The scale corresponded to the following meanings: 1 indicated that the item does not meet the criterion, 2 signified a low level of compliance, 3 represented a moderate level of compliance, and 4 indicated a high level of compliance [31]. Empirical techniques were then applied to calculate the content validity index (CVI), the content validity ratio (CVR), and the modified Kappa agreement index (K) [32,33].

The content validity index (CVI) quantifies the content validity of multiple-item scales based on expert ratings regarding the relevance of each item. This indicator is determined by the proportion of experts who rated an item with a score of 3 or 4 compared to the total number of experts who assessed that item in terms of clarity and relevance [32]. The scale content validity index (S-CVI) is used to assess validity by scale, which is derived from the proportion of items that received a score of 3 or 4 in terms of clarity and relevance from at least 80% of the experts [34]. The item content validity index (I-CVI) is employed to evaluate each feature within the scales, calculated as the proportion of experts who rated 3 or 4 divided by the total number of experts who rated that item [32]. An I-CVI value of ≥0.83 is deemed acceptable. Additionally, universal agreement among experts (S-CVI/UA) and average agreement (S-CVI/Ave) were computed, with values of S-CVI/UA ≥ 0.83 and S-CVI/Ave ≥ 0.9 considered satisfactory [29]. To calculate the modified Kappa statistic, first, the probability of chance agreement is calculated for each element through the following formula: K = (I-CVI − Pc)/(1 − Pc), where Pc = [N!/A! (N − A)!] * 0.5 N. In this formula, Pc is the probability of chance agreement, N is the number of experts, and A is the number of experts who agree that the item is relevant. Kappa values above 0.74 are considered excellent [32].

The qualitative data for content validity were obtained from all the comments provided by the experts in the observations section of the evaluation rubric.

The entire validation process was carried out in two stages. In the first stage, the experts evaluated the material in terms of face and content, after which the research team made the changes suggested based on their observations. Subsequently, in the second stage, the experts were asked to confirm whether these modifications appropriately addressed the recommended adjustments.

### 2.4. Stage 4: Pilot Test Procedure

Following the cross-cultural adaptation and validation, both for face and content, a pilot test was conducted to obtain a final version. For the pilot test, 10 new experts were selected through convenience sampling (7 women and 3 men), all working in different healthcare centers in Chile, providing care to patients with speech neuromotor disorders.

In this case, the K coefficient was not calculated, as the primary interest at this stage was the participants’ clinical experience in assessing subjects with dysarthria rather than their status as experts from an academic perspective. For this reason, the language-speech pathologists who participated in this phase were not the same as those in the previous stages.

Once the instrument was adapted and validated, each expert was required to assess a patient with dysarthria. Following this, the participating speech–language pathologists had to complete a cognitive interview. Through this interview, the following aspects were explored: (1) their overall impression of the BoDyS, (2) their opinion regarding the instructions of the BoDyS-CL, (3) their insights on the feasibility of applying the BoDyS-CL, (4) their perspectives on the content of the BoDyS-CL, and (5) their evaluation of the tasks in the BoDyS-CL. Based on the analysis of the experts’ feedback, final modifications were made to the instrument’s final version.

## 3. Results

### 3.1. Stage 1: Findings from Cross-Cultural Adaptation

In this stage, based on the translation and back-translation procedures, some technical terms used in Chile to describe dysarthria characteristics were adjusted in the ‘Respiration during Speech’ scale (‘respiration’ in the original version) and the ‘Vocal Quality’ scale (‘vocal quality’ in the original version). Additionally, a clinical description was incorporated for each feature to guide clinicians and provide greater clarity on how to identify them during clinical assessment. This process resulted in the agreed-upon and adapted version of the description of the scales and their features.

**Figure 2:**
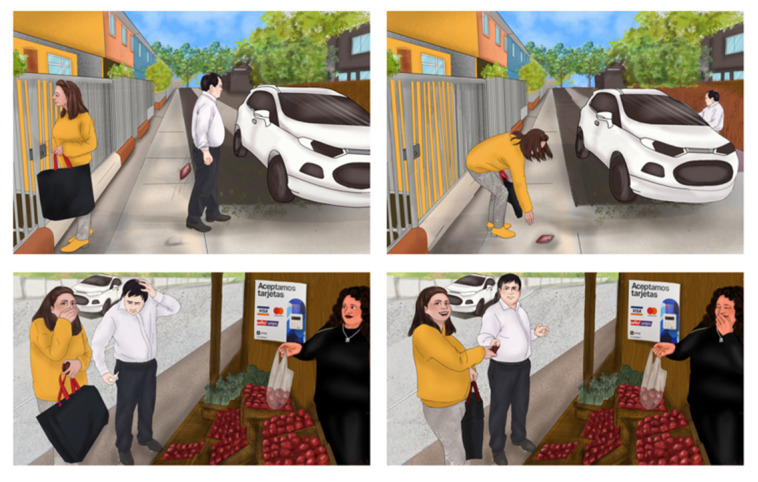
Picture No. 2 for the description of BoDyS-CL.

Regarding the linguistic adaptations of the stimuli in the Chilean Spanish version, the following changes were made. (1) In the “Spontaneous Language” task, which consists of 12 questions to request a speech sample, the questions were adapted following the rules of the original scale in terms of semantic accuracy, length, and complexity. (2) In the “Sentence Repetition” task, which consists of 15 sentences, these were adapted following the rules of the original scale regarding lexical, phonological, length, and complexity aspects. (3) An example of the sentences in German and Spanish, respectively, is “What is your profession?” (translated from German), with the Chilean adaptation being “What is your occupation?”. The “Reading” task, consisting of three new texts, was created following the rules of the original scale regarding semantic accuracy, length, and complexity. (4) In the “Picture Description” task, which consists of three new sequences of four images each, they were created following the rules of the original scale regarding the number of images and complexity (Figure 2).

### 3.2. Stage 2: Face Validation Results

Experts for this study stage were selected through a questionnaire that assessed their expertise in the relevant area, employing the K coefficient for expert selection [27]. Those experts who achieved a score higher than 0.8 were included, as their level of knowledge was deemed sufficient. A group of 10 experts were engaged in this process, which was conducted in two rounds to allow the experts to review the changes made after the first round and to facilitate a new validation process.

The face validation focused on specific sections of the BoDyS: the stimuli manual, the descriptions of the scales and features, and the recording sheet. Each section was evaluated based on clarity, length, and format using a 5-point Likert scale. In the first round, the experts evaluated the face of the instrument as follows:

In the evaluation of the stimuli manual, regarding the criterion of clarity, 30% of the experts suggested adjusting the instruction for the task “Spontaneous Language Instruction,” while the remaining 70% fully agreed with the clarity of the instructions. Regarding the format criterion, 90% of the experts were completely satisfied with the presentation of the information, images, font, and/or font size. However, one expert suggested increasing the font and image sizes to improve visibility for potential patients. Regarding the length of the manual, 90% of the experts indicated that the length of the stimuli was appropriate and allowed for reading comprehension. However, one expert mentioned that the number of tasks might be excessive for patients who experience higher fatigability. It is important to note that the original version of BoDyS includes a reduced version with fewer tasks to mitigate fatigue effects. Nevertheless, this study was conducted using the full version.

Regarding the scales’ description and features, 80% of the experts stated that the text is clear and contains appropriate semantic and syntactic aspects. Similarly, 80% of the experts indicated that the format, in terms of information presentation, images, font, and/or font size, is adequate. Regarding the length of this section, 90% of the experts indicated that the “length of the texts is appropriate and allows for reading comprehension.” However, one expert mentioned that the description of the features might be too extensive to cover within a single assessment session.

Regarding the recording sheet, where the frequency of occurrence of dysarthria characteristics—or features—is recorded for each scale, the entire group of experts fully agreed that the text contains appropriate semantic, syntactic, and length-related aspects concerning the criteria of clarity and length. Concerning the format criterion, 20% of the experts indicated that it would be necessary to include a summary table of performance scores to guide the process of constructing the dysarthria profile.

Based on the experts’ evaluation, the following changes were introduced in the recording sheet: (1) the instruction on how to record the features was incorporated, (2) a table summarizing the total task scores for the scales was added, and (3) a table for constructing the dysarthria profile was included. Similarly, based on the experts’ feedback, the following modifications were made to the stimuli manual: (1) the font size of the stimuli was increased, and (2) numbering was added to the image sequences to facilitate their description.

During the second round of face validation, unanimous agreement was achieved for clarity, length, and format.

### 3.3. Stage 3: Content Validation Results

This process involved validating the description section of the scales and their corresponding features to assess the clarity and relevance of each feature, as well as the adequacy of the scales. Both qualitative and quantitative analyses of the results were conducted. For the qualitative analysis, all expert comments were critically examined. Based on this analysis, the experts suggested the only modification to the ‘Vocal Characteristics During Speech (REG)’ scale, changing feature 3 and feature 4 from ‘very loud volume’ and ‘very soft volume’ to ‘increased volume’ and ‘decreased volume’, respectively.

On the quantitative side, regarding the I-CVI indicator, which was applied to evaluate the clarity and relevance of the scale items, there was absolute agreement among the evaluators (I-CVI = 1.00) for each feature in every scale. This indicates that all the evaluated features were considered clear and relevant.

For the S-CVI indicator, which was applied to quantify the clarity and relevance criteria of the scale in general and used to evaluate each of the scales, the values obtained were 1.00 for both the average agreement (S-CVI/Ave) and the universal agreement among experts (S-CVI/UA) for the two evaluated criteria of clarity and relevance. In this case, following Zamanzadeh’s criteria [29], high levels of agreement among experts were observed in the evaluation of these criteria.

The Kappa index was calculated based on the clarity and relevance criteria. In both review rounds, all Kappa indexes were equal to 1, indicating an excellent level of agreement.

The CVR index was calculated to determine the relevance of each item. In both rounds, values of 1.00 were obtained, exceeding the previously established thresholds [29,30,35] for acceptability. As a result, all items were considered essential for the instrument.

After completing the cross-cultural adaptation and the face and content validation processes, a pilot test was conducted to finalize the Chilean Spanish-adapted version of the BoDyS.

### 3.4. Results from the Pilot Test

Ten expert speech–language pathologists assessed a patient with dysarthria using the adapted and validated version of the BoDyS. Following this, each professional participated in a cognitive interview to identify potential difficulties in applying the instrument and to understand the clinician’s experience during the process.

The information gathered from the interviews was systematically organized, yielding the following results:

Concerning the general impression of the cross-culturally adapted Chilean Spanish version of the BoDyS (BoDyS-CL), the interviewees noted that the test provides accurate information about the patient’s performance and is easy to apply. However, they pointed out that the application time could be lengthy, which may cause patients with more severe impairments to fail to complete the test due to fatigue.

Regarding the instructions of the BoDyS-CL, the interviewees remarked that the guidelines for each task are clear, and all experts unanimously agreed that no changes are necessary.

In terms of the feasibility of applying the BoDyS-CL, the interviewees indicated that the organization, instructions, and format of the assessment tasks are appropriate. However, they suggested improving the clarity and size of images used in the picture sequence description task.

Concerning the content of the BoDyS-CL, interviewees reported that the instrument is highly comprehensible, and the instructions are appropriate, with no need for rewording. They highlighted that the open-ended question task to elicit spontaneous speech is the most effective way of assessing the patient’s speech performance. However, they also pointed out that the complete application of the test is lengthy for patients, and they did not see the need to repeat the tasks three times, as the original full version of the test requires.

The opinions regarding the tasks of the BoDyS-CL were generally positive, with the instrument regarded as sufficient. However, there is a highlighted need for tasks that allow for the determination of the neuromotor performance of speech structures and the necessity of evaluating the fundamental motor processes of articulation in more detail. This approach would enable a more accurate characterization of altered speech sounds.

Based on the evaluation, it was decided to include more detailed information regarding test scoring in the instruction manual. With all the processes outlined here, the Chilean version of the BoDyS is considered both adapted and validated.

## 4. Discussion

The cultural adaptation and content validation process for the BoDyS, which evaluates speech in individuals with dysarthria and was initially developed in German, was carried out using exhaustive and iterative processes. The final version includes 9 scales describing different dimensions of speech, along with a stimuli manual containing 12 speech tasks. The Chilean Spanish adapted scales were critically evaluated for face and content validity through a judgment from 10 experts. Following this, they were subjected to a pilot test by 10 professionals with experience working with individuals with dysarthria.

While the direct translation process for the cross-cultural adaptation progressed without issues, a “clinical description” section was added to the scale descriptions of the original version for each of the features per scale. This addition aimed to assist evaluators and provide more precise guidance on identifying speech impairments during assessments. As a result, a consensus was reached on the adapted versions of the scale descriptions and their associated features.

As a result of the observations made by the experts regarding face validity, modifications were made to the instrument format, including an increase in the size of the stimuli and the font. These changes were implemented due to concerns about their potential impact on patients’ performance. This adjustment considers that dysarthria is more prevalent among older adults, a demographic that is at a higher risk of visual impairments, such as age-related macular degeneration (AMD). This condition significantly decreases quality of life, particularly affecting the ability to read, recognize faces, and drive [36]. The most recent National Disability and Dependency Survey in Chile [37] reports that 44% of individuals over 60 years old experience some degree of visual impairment, with 42% experiencing partial vision loss and 2% being entirely blind. Additionally, dysarthria is commonly associated with Parkinson’s disease, which can also lead to visual impairments, such as reduced eye movement and difficulty distinguishing visuospatial aspects. Even with visual stimuli, Parkinson’s patients may have difficulty processing certain information [38]. It is estimated that 82% of individuals with Parkinson’s disease experience at least one ophthalmological symptom [39]. It is worth noting that the original BoDyS version provides standard norms for a test variant that excludes reading tasks, thus supporting the assessment of patients with visual (and other) reading difficulties [20].

Another modification implemented was the addition of numbering to the images used in storytelling. This measure was introduced to reduce cognitive load, particularly considering some patients may experience dysarthria alongside language or cognitive impairments, which could impact assessment outcomes [40,41]. The intention behind this scale is not to increase the cognitive effort needed to follow the sequence of images but rather to ensure that patients can comprehend the images and narrate a story fluently, thereby enabling the collection of a meaningful speech sample. In older adults, there is a decrease in cognitive and motor processing speed [42]. Therefore, it is crucial to consider the effects of this slowdown on performance in various cognitive tasks when interpreting impairment profiles in this population, particularly by distinguishing between the speed and quality of performance across different activities. Additionally, it is essential to note that patients who have suffered a stroke frequently experience an immediate decline in their cognitive abilities, including thinking and reasoning skills, as well as a gradual deterioration in mental processes over time. These patients have a higher likelihood of experiencing accelerated deterioration in their cognitive and planning abilities for at least six years following the medical incident [43,44]. This aspect must be considered when assessing patients with neuromotor disorders, such as dysarthria, and their performance in speech-related tasks.

On the other hand, modifications aimed at improving clarity focused on how to track results. Experts noted that the original instructions were insufficiently precise, potentially leading to errors in analysis and incorrect diagnoses. Proper record-keeping enables more accurate result analysis, thereby enhancing decision-making for more effective therapeutic interventions [45]. Furthermore, accurate records facilitate data collection that can be compared with future assessments, supporting more rigorous monitoring of the patient’s progress.

Regarding task length, some experts have noted that repeating tasks three times may be excessively tiring for specific patients with dysarthria. This insight stems from the understanding that, depending on the underlying cause of dysarthria, a range of cognitive or motor characteristics may be present. These characteristics can vary based on the level and severity of the lesion, potentially affecting the central nervous system, the peripheral nervous system, or both [22]. For instance, patients may experience cognitive fatigue, particularly those with hypokinetic dysarthria associated with Parkinson’s disease. Cognitive impairments in this type of dysarthria are primarily evident in executive functions and visuospatial skills, accompanied by a marked decline in information processing speed, which can hinder performance during extended assessments [46]. On the other hand, in other cases, fatigue can have a significant impact at the motor level, as seen in myasthenia gravis, which affects the neuromuscular junction and causes an inability of muscles to maintain the strength of their contractions over time, leading to rapid fatigue and muscle weakness [47]. However, it is important to highlight that the BoDyS have abbreviated versions, consisting of eight speech samples—that is, two rounds of tasks—specifically designed for patients with greater fatigability and attentional difficulties. Additionally, there is a non-reading version that includes a total of nine speech samples and excludes all tasks involving reading.

These versions which will be evaluated and validated in a subsequent stage. The availability of abbreviated formats will facilitate more appropriate assessments for patients experiencing either motor or cognitive fatigue.

Regarding the content validity process, the scale that received the most suggestions from the expert panel was the ‘vocal characteristics during speech’ scale, particularly the ‘volume’ feature, where the terms ‘very soft’ and ‘very loud’ were replaced with ‘increased’ and ‘decreased’. This consensus and subsequent modification were based on the technical language used in the existing dysarthria literature, where the concepts of increase and decrease describe variability in tone and volume [1]. In terms of the quantitative content validity results, both the first and second rounds were deemed adequate in clarity, relevance, and sufficiency, meeting the necessary criteria to proceed to the pilot phase.

The pilot phase allowed for identifying potential new difficulties during the application of the instrument and for gaining insights into the clinician’s experience throughout the process. The experts indicated that the test provides accurate information about the patient’s performance, is easy to apply, and provides clear instructions. This aspect is particularly significant, as it enhances the validity and reliability of the results, minimizing the risk of errors that could compromise diagnostic accuracy [45]. It also reduces inter-observer variability, which is crucial for maintaining assessment consistency and improving clinical outcomes [48]. Furthermore, the clarity of the instructions contributes to operational efficiency, allowing procedures to be carried out more smoothly with fewer clarifications or repetitions. Evidence suggests that clear protocols reduce the time required to complete assessments without compromising their accuracy [49].

Among the various tasks in the assessment, the “Spontaneous Speech” task stands out as it offers a more comprehensive understanding of speech performance. This task provides valuable insights into how patients communicate in real-life situations, an aspect that structured or repetition tasks cannot fully capture. The inherent variability of spontaneous tasks more accurately reflects the challenges patients face daily, leading to a more representative assessment [50]. Furthermore, it is important to note that spontaneous speech tasks require the integration of the subcomponents of speech, allowing the clinician to observe how these systems interact during natural speech production. This perspective is supported by studies such as those conducted by Dos Santos et al. [51] or Brendel et al. [52], indicating that spontaneous speech tasks deliver a broader view of a patient’s neuromotor speech capabilities. However, experts generally mentioned that repeating the tasks three times results in an overly long application time, during which older patients and/or those with neurological impairments may experience cognitive and motor fatigue, thus preventing them from completing the test. It is worth noting that these tasks do not necessarily need to take much time. Each task consists of only one topic/question, and the examiner may decide to end the interaction as soon as she/he has picked up enough of the patient’s speech symptoms in her/his response. This characteristic is essential to prevent patient fatigue when taking the test; however, due to the nature of the validation process, the judges had to familiarize themselves with the full instrument rather than its shortened versions to ensure the availability of the complete Spanish version.

Additionally, experts highlighted the need for this scale to include tasks assessing the neuromotor performance of the anatomical structures involved in speech. Duffy [2] emphasizes the importance of a detailed understanding of anatomical alterations when accurately identifying the source of speech difficulties in patients with dysarthria. These alterations can impact speech production in various ways, ranging from muscular weakness to coordination issues in speech movements. Conversely, Feenaughty (2021) [41] points out that utilizing detailed clinical assessments allows for a more precise assessment of the orofacial structures, which in turn facilitates the customization of treatments to meet each patient’s specific needs.

Given the importance of accurate diagnosis and precise monitoring during the therapeutic management of dysarthria, the BoDyS can potentially improve clinical decision-making, therapeutic planning, and outcome monitoring in the Chilean healthcare context by providing comparable measures of patient performance over time [53].

While this study presents valuable contributions, several limitations must be acknowledged. The sample size was small, which may affect the representativeness of the results. However, a notable degree of consensus was observed among the judges during both rounds of the Delphi process. It is important to recognize that since the adaptation is specifically for Chilean Spanish, all judges were Chilean, which may restrict its applicability to other Spanish variants. Additionally, it is essential to note that content and face validation do not assess the instrument’s performance in actual clinical practice, thus not ensuring its effectiveness in assessing patients with dysarthria. Therefore, future research should complement this phase with reliability studies that examine the scales’ performance in pilot tests with patients, including test–retest reliability and inter-rater consistency, to ensure the instrument’s stability over time and across evaluators. Also, comparisons of the instrument’s results with objective measures of speech and across different populations with dysarthria should also be included to verify its effectiveness in varied clinical and linguistic contexts.

Overall, the BoDyS should be supplemented to ensure a comprehensive assessment of a patient’s performance, as it currently overlooks aspects related to the patient’s quality of life and does not include neuromotor or orofacial assessments. These elements are essential for providing more precise insights into the dysarthria level and type, as recommended by ASHA [8]. Therefore, it is suggested that the BoDyS be associated with additional instruments, such as orofacial motor examinations and the QOL-DyS, a self-assessment questionnaire that measures health-related quality of life in individuals with dysarthria [54]. Including such complementary assessments would enhance the clinical utility of the BoDyS and provide a more holistic understanding of the patient’s communicative functioning.

## 5. Conclusions

The cross-cultural adaptation and validation of the BoDyS to Chilean Spanish provides a valuable tool for assessing dysarthria within the Chilean context. The findings indicate that this adapted version is clear and relevant in its instructions, content, and format, making it suitable for assessing the functional aspects of speech. However, experts identified areas for improvement, such as task duration and the need for supplementary assessment of neuromotor and orofacial functions.

This study provides a foundation for the systematic implementation of the adapted BoDyS in clinical practice in Chile. It paves the way for future research that may include the assessment of other relevant dimensions in dysarthria treatment. Ultimately, this tool aims to facilitate the development of more effective and personalized therapeutic interventions, thereby enhancing the quality of life for patients with dysarthria.

## Figures and Tables

**Table 1:** BoDyS and their features.

Scale	Features
RSP (respiration)	Frequent inspirations Audibly effortful inspirations Expiration beyond the resting level
VOL (voice level)	High-pitch Low-pitch Loud voice Soft voice
VOQ (voice quality)	Breathy and harsh Strained–strangled and harsh
VOS (voice stability)	Fluctuations of pitch, loudness, and/or quality Voice breaks and voice fading Voice tremor Involuntary vocalizations
ART (articulation)	Reduced Overshooting Wide Narrow Variable
RSN (resonance)	Hypernasality and nasal emission Hyponasality Intermittent hyper- or hyponasality
TEM (articulation rate)	Decreased articulation rate Increased articulation rate
FLU (fluency)	Pauses Iterations
MOD (prosodic modulation)	Reduced prosodic modulation of pitch and/or loudness Excessive prosodic modulation of pitch and/or loudness Conspicuous rhythm and/or stress patterns

Note: Referenced based on the proposal by Ziegler et al. (2017) [20].

## Data Availability

The data supporting the conclusions of this article cannot be provided due to the sensitivity of the data collected during the study.
